# Measurement of Aβ Amyloid Plaques and Tau Protein in Postmortem Human Alzheimer’s Disease Brain by Autoradiography Using [^18^F]Flotaza, [^125^I]IBETA, [^124/125^I]IPPI and Immunohistochemistry Analysis Using QuPath

**DOI:** 10.3390/biomedicines11041033

**Published:** 2023-03-27

**Authors:** Rommani Mondal, Yasmin K. Sandhu, Vallabhi M. Kamalia, Brooke A. Delaney, Amina U. Syed, Grace A. H. Nguyen, Taylor R. Moran, Roz R. Limpengco, Christopher Liang, Jogeshwar Mukherjee

**Affiliations:** Preclinical Imaging, Department of Radiological Sciences, University of California-Irvine, Irvine, CA 92697, USA

**Keywords:** [^18^F]Flotaza, [^125^I]IPPI, [^124^I]IPPI, [^125^I]IBETA, Alzheimer’s disease, Aβ amyloid plaques, tau, neurofibrillary tangles, QuPath, PET imaging, autoradiography

## Abstract

High-resolution scans of immunohistochemical (IHC) stains of Alzheimer’s disease (AD) brain slices and radioligand autoradiography both provide information about the distribution of Aβ plaques and Tau, the two common proteinopathies in AD. Accurate assessment of the amount and regional location of Aβ plaques and Tau is essential to understand the progression of AD pathology. Our goal was to develop a quantitative method for the analysis of IHC–autoradiography images. Postmortem anterior cingulate (AC) and corpus callosum (CC) from AD and control (CN) subjects were IHC stained with anti-Aβ for Aβ plaques and autoradiography with [^18^F]flotaza and [^125^I]IBETA for Aβ plaques. For Tau, [^124^I]IPPI, a new radiotracer, was synthesized and evaluated in the AD brain. For Tau imaging, brain slices were IHC stained with anti-Tau and autoradiography using [^125^I]IPPI and [^124^I]IPPI. Annotations for Aβ plaques and Tau using QuPath for training and pixel classifiers were generated to measure the percent of the area of Aβ plaques and Tau in each slice. The binding of [^124^I]IPPI was observed in all AD brains with an AC/CC ratio > 10. Selectivity to Tau was shown by blocking [^124^I]IPPI with MK-6240. Percent positivity for Aβ plaques was 4–15%, and for Tau, it was 1.3 to 35%. All IHC Aβ plaque-positive subjects showed [^18^F]flotaza and [^125^I]IBETA binding with a positive linear correlation (r^2^ > 0.45). Tau-positive subjects showed [^124/125^I]IPPI binding with a stronger positive linear correlation (r^2^ > 0.80). This quantitative IHC–autoradiography approach provides an accurate measurement of Aβ plaques and Tau within and across subjects.

## 1. Introduction

Alzheimer’s disease (AD) is characterized by the accumulation of Aβ plaques and neurofibrillary tangles (NFT) of Tau protein in the brain [1]. Postmortem human brain studies are an important step toward understanding the role of biomarkers in disease progression, the interrelationships between different biomarkers and regional biomarker differences in different parts of the brain. Immunohistochemical (IHC) studies have been used to evaluate levels of Aβ plaques and NFT [2]. High-resolution scans of IHC stains of AD brain slices and radioligand binding to Aβ plaques and Tau both give us information about the detailed tissue distribution of these two neuropathologies. Accurate assessment of the amount and regional location of Aβ plaques and Tau is essential to understand AD progression and may provide additional insights for in vivo PET imaging [3,4,5].

Although a visual inspection of autoradiography and IHC images at first glance provides information about the localization of Aβ plaques and Tau, a quantitative/semi-quantitative approach for comparison of the two techniques is essential. Several approaches have been reported for measuring Aβ plaque load and Tau load using IHC brain sections of AD [6]. These include measuring the intensity of staining and measuring Aβ plaque load as a percent of Aβ plaque positivity [7]. Digital analysis for quantification of Aβ plaques has been reported for neuropathological assessment as a rapid and automated method of analysis of multiple samples [8]. In radioligand autoradiographic studies, the use of IHC stains has been qualitative to confirm the presence of Aβ plaques or Tau in corresponding regions of the brain specimens [9,10,11,12,13,14]. However, a quantitative/semi-quantitative comparison of the binding of radioligand with IHC is necessary in order to validate the measurements. Toward understanding the pathological effects of the accumulation of Aβ plaques and Tau on the various neurotransmitter receptor systems [15], it is vital that an accurate, quantitative assessment of Aβ plaques and Tau using radioligand-IHC is available.

Imaging agents for both Aβ plaques and NFT are currently being used in human PET studies to understand the timeline of the two proteinopathies in the aging brain of AD subjects [3]. We have reported the use of [^18^F]flotaza [10] and [^125^I]IBETA [11] for imaging Aβ plaques and [^125^I]IPPI for imaging Tau [9]. Our previous work on [^18^F]flotaza, [^125^I]IBETA and [^125^I]IPPI involved quantitation of the autoradiographic images using the Optiquant Imaging System. This allowed for the quantitative measurement of radiotracer binding in terms of digital light units/mm^2^ (DLU/mm^2^) in different brain sections and regional differences within brain slices and across different subjects. The measured amount of radiotracer binding was interpreted as the amount of Aβ plaque load for [^18^F]flotaza [10] and [^125^I]IBETA [11] and the amount of Tau for [^125^I]IPPI [9]. These measures were qualitatively compared with IHC staining for anti-Aβ and anti-Tau for regions of high and low binding. These comparisons of the binding of the radiotracers were made across different subjects. A more quantitative correlation of [^18^F]flotaza and [^125^I]IBETA for imaging Aβ plaques and [^125^I]IPPI for Tau with anti-Aβ and anti-Tau IHC is now desired. This will allow a more accurate analysis of the binding of the radioligand to the various forms of Aβ plaques (neuritic, cored or diffused [16,17]) as well as the different forms of Tau [18,19].

**Figure 1:**
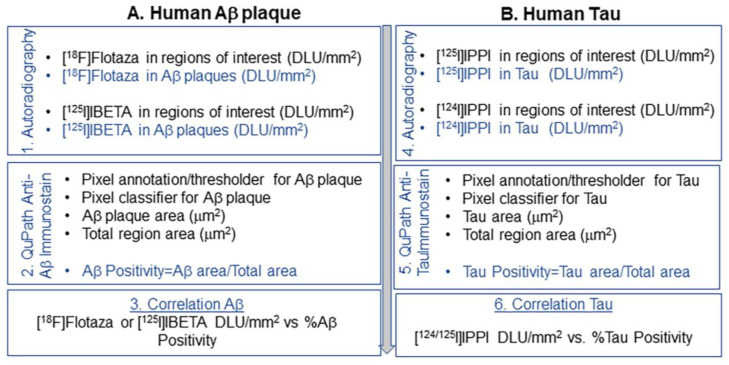
Sequence of steps followed for image analysis of (**A**). Aβ plaques and (**B**). Tau: (1). [^18^F]Flotaza and [^125^I]IBETA autoradiography were used to measure Aβ plaques in digital light units, DLU/mm^2^ in brain sections; (2). IHC analysis using QuPath measures Aβ positivity per region of interest; (3). Quantitative correlation of [^18^F]flotaza and [^125^I]IBETA per mm^2^ with Aβ positivity per region of interest; (4). [^124/125^I]IPPI autoradiography measured for Tau in DLU/mm^2^ in brain sections; (5). IHC analysis using QuPath measures Tau positivity per region of interest; (6). Correlation of [^124/125^I]IPPI per mm^2^ with Tau positivity per region of interest.

Thus, our goal was to quantitatively assess IHC images of Aβ plaques and Tau using the digital pathology image analysis software QuPath [20] and correlate them with radioligand autoradiography. Figure 1 describes the steps involved in the approach. Using QuPath, Aβ plaque load was computed as %Aβ positivity=area of Aβ plaques in the region of interest/total area of the region of interest. Similarly, Tau load was computed as %Tau positivity=area of Tau in the region of interest/total area of the region of interest. Quantitative information of [^18^F]flotaza and [^125^I]IBETA were then correlated with %Aβ positivity, and [^125^I]IPPI was correlated with %Tau positivity in each subject. The quantitative measures of IHC and radioligand binding of Aβ plaques could then be compared with Tau in order to ascertain correlations between the two proteinopathies. The anterior cingulate cortex has been shown to be afflicted with both Aβ plaques and Tau in the AD brain [21]. Our previous radioligand studies have confirmed the accumulation of [^18^F]flotaza [10], [^125^I]IBETA [11] and [^125^I]IPPI [9] in this brain region. Since iodine-124 is a useful PET radionuclide with the added advantage of a longer half-life compared to other PET radioisotopes such as carbon-11 and fluorine-18 [11,13], we have now prepared [^124^I]IPPI as a PET radiotracer analog of [^125^I]IPPI. Here, we report the radiosynthesis and in vitro evaluation of [^124^I]IPPI and quantitative correlation of [^18^F]flotaza, [^125^I]IBETA and [^124^/^125^I]IPPI radioligand binding with IHC in the anterior cingulate cortex of six well-characterized, cognitively normal control subjects (CN) and six AD subjects.

## 2. Materials and Methods

### 2.1. General Methods

Fluorine-18 labeled [^18^F]Flotaza [10], [^125^I]IBETA [11] and iodine-125 labeled [^125^I]IPPI [9] were prepared as reported previously. Capintec CRC-15R dose calibrator and Capintec Caprac-R well-counter (Florham Park, NJ, USA) were used for radioactivity measurements. Thin layer chromatography of radioligands was scanned on an AR-2000 imaging scanner (Eckart and Ziegler, Berlin, Germany). Cyclone phosphor autoradiographic imaging system (Perkin Elmer Inc, Shelton, CT, USA) and Optiquant Imaging System software were used for analysis. Immunostaining of brain sections was carried out by UCI Pathology core services. QuPath (version QuPath—0.2.3) was used for quantitative analysis of scanned brain slices.

### 2.2. Radiosynthesis of [^124^I]IPPI

**Figure 2:**
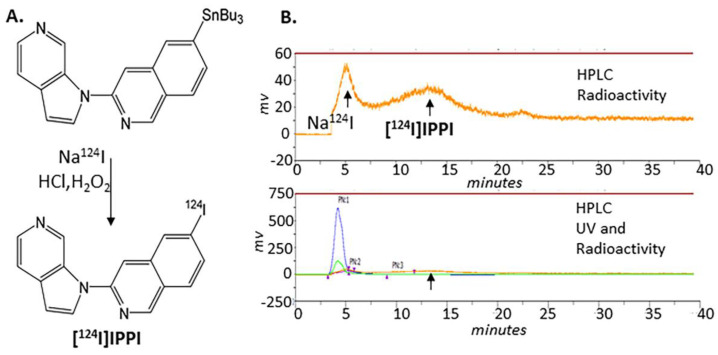
Radiosynthesis scheme of [^124^I]IPPI. (**A**). Tributyltin precursor was reacted with sodium [^124^I]iodide to give [^124^I]IPPI. (**B**). Reaction mixture was purified on reverse-phase HPLC, and pure [^124^I]IPPI product was collected at retention time between 10 and 15 min (top panel shows radioactivity profile, and bottom panel shows accompanying UV profile).

Using sodium iodide, [^124^I]NaI (3D Imaging LLC, Little Rock, AR, USA), [^124^I]IPPI was prepared by electrophilic substitution of the tributyltin derivative (Figure 2) using our previously reported radioiodination methods [11,13]. Tributyltin azaindole derivative (Figure 2A, [9], 0.1 mL of 1 mg/0.2 mL of ethanol), 21 MBq [^124^I]NaI and 0.1 mL of 1N HCl were taken in a vial followed by the addition of 0.1 mL H_2_O_2_ (3%). The mixture reacted at room temperature for 30 min and was terminated by the addition of sodium bisulfite. The crude [^124^I]IPPI was purified by HPLC on a semi-preparative column (60% acetonitrile:40% 0.1% triethylamine). Retention time of [^124^I]IPPI was 13 min, with no significant levels of UV peaks with the product (Figure 2B). The collected fraction of [^124^I]IPPI was found to be >95% pure by Radio TLC. Specific activity was estimated to be >500 TBq/mmole.

### 2.3. Molecular Models

The binding of the radiotracers to Aβ plaques and Tau was assessed using published cryo-electron microscopy models of Aβ1−42 and Tau. We used Chimera software with Autodock Vina for interactive visualization and analysis of molecular structures [9]. Autodock Vina provides binding site identification and binding conformation-free energies in ligand–protein integration [22]. 

**Figure 3:**
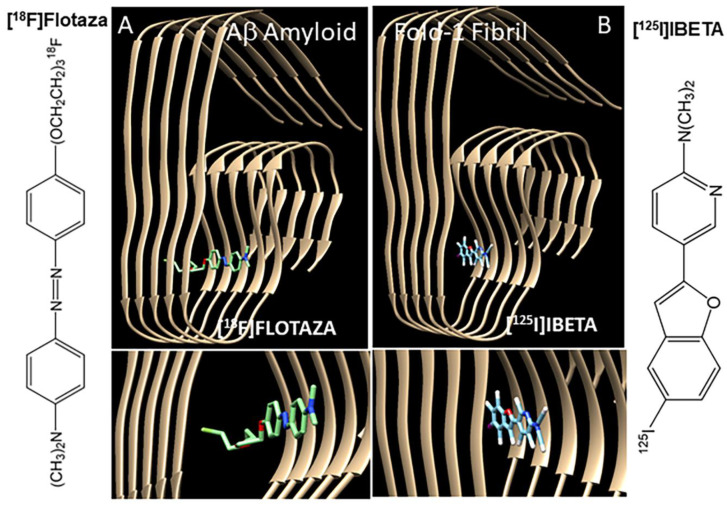
Aβ fibril model with flotaza and IBETA: Chimera–Autodock Vina models of Aβ amyloid fold-1 fibril showing flotaza (**A**) and IBETA (**B**) binding sites. Preferred binding sites of [^18^F]flotaza (**A**) and [^125^I]IBETA (**B**) to the Aβ fibril were similar. The measured binding energy of [^18^F]flotaza was −8.7 kcal/mol, and for [^125^I]IBETA, it was −11.5 kcal/mol.

The cryo-electron microscopy (cryo-EM) fibril structure of Aβ [23] was used for assessing binding of flotaza and IBETA. Previous studies have evaluated structure of Aβ 1-42 fibril using small molecule binding [24,25]. The molecular structures (mol2 files) of flotaza and IBETA were built using the ChemDraw software (Ithacus software, Inc), and a blind docking with chosen grid box was selected to perform molecular docking. The grid box dimensions were chosen so that the surface sites in the Aβ amyloid fibril were captured (Figure 3). The clusters corresponding to binding energies (represented by Kcal/mol) with the lowest negative scores were used. 

**Figure 4:**
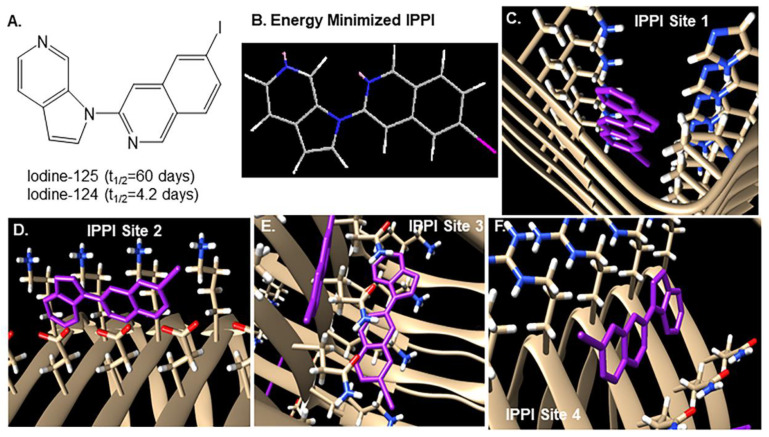
Tau model with IPPI: (**A**). Chemical structure of IPPI; (**B**). Energy minimized structure of IPPI used in Chimera–Autodock. (**C**–**F**). IPPI docked in four different sites in the cryo-EM model of Tau using the Chimera–Autodock Vina. The binding energies of IPPI were as follows: Site 1= −7.8, Site 2= −8., Site 3= −8.2 and Site 4= −7.5 kcal/mol [9].

Cryo-EM three-dimension (3D) paired helical filament (PHF) structure of Tau fibril was used to perform molecular prediction on the Tau fibril (Figure 4). Blind docking of IPPI was performed at four different binding sites, as previously reported [9,26].

### 2.4. Subjects

Human postmortem brain tissue samples of AD and CN subjects (male and female), each consisting of anterior cingulate cortex and corpus callosum, were obtained from Banner Sun Health Research Institute, Sun City, AZ, USA, brain tissue repository for in vitro experiments (Table 1). Brain slices, 10 μm thick, were obtained from the chunks of frozen tissue on a Leica 1850 cryotome cooled to −20 °C and collected on Fisher slides. All slides were then stored at −80 °C. All postmortem human brain studies were approved by the Institutional Biosafety Committee of University of California, Irvine. 

### 2.5. Immunohistochemistry

All adjacent brain slices were immunostained for Tau and Aβ plaques. For total Tau, DAKO polyclonal antibody, which detects all 6 six isoforms of Tau, was used at a dilution 1: 3000, A0024 (Agilent, Santa Clara, CA, USA) using reported protocols [27]. Brain slices from all subjects were immunostained with anti-AβBiolegend 803,015 (Biolegend, San Diego, CA, USA), which is reactive to amino acid residue 1–16 of β-amyloid. Anti-Tau and anti-Aβ immunostained slides were scanned using the Ventana Roche slide scanner, and the images generated were used for analysis by QuPath.

### 2.6. Autoradiography

#### 2.6.1. [^18^F]Flotaza for Aβ

Brain sections were treated with [^18^F]Flotaza (740 kBq/mL) using previously reported methods [10]. Dried radiolabeled brain sections were exposed overnight on a phosphor film, and the films were read on the Phosphor Autoradiographic Imaging System (Packard Instruments Co, Boston, MA, USA). Regions of interest (ROIs) were drawn on the slices, and the extent of binding of [^18^F]Flotaza was measured using the OptiQuant acquisition and analysis program.

#### 2.6.2. [^125^I]IBETA for Aβ Plaques

Brain sections were treated with [^125^I ]IBETA (5 kBq/mL) using previously reported methods [11]. The brain sections were air dried, exposed for 7 days on a phosphor film and then placed on the Phosphor Autoradiographic Imaging System (Packard Instruments Co). Regions of interest (ROIs) were drawn on the slices, and the extent of binding of [^125^I]IBETA was measured using the OptiQuant.

#### 2.6.3. [^125^I]IPPI and [^124^I]IPPI for Tau

Brain sections were treated with [^125^I]IPPI (60 mL; 3.7 kBq/mL) or with [^124^I]IPPI (60 mL; 6 kBq/mL) in 10% ethanol PBS buffer pH 7.4. The chambers were incubated at 25 °C for 1.25 h, as reported [9]. The brain sections were air dried and exposed for 24 h on a phosphor film for iodine-124 due to its higher photon energy and half-life or 7 to 14 days for iodine-125 due to its lower photon energy and longer half-life. The films were then read on the Phosphor Autoradiographic Imaging System/ Cyclone Storage Phosphor System (Packard Instruments Co). Regions of interest (ROIs) were drawn on the slices, and the extent of binding of [^125^I]IPPI and [^124^I]IPPI was measured in DLU/mm^2^ using the OptiQuant.

### 2.7. Optiquant Image Analysis

Anterior cingulate and corpus callosum were the two major regions of interest (ROIs) that were drawn on autoradiographs using Optiquant. Optiquant gave measures of digital light unit (DLU) per unit mm^2^ area from the pixels of an autoradiographic image. Background activity levels were subtracted from all images. Higher DLU/mm^2^ from autoradiography indicated higher [^18^F]flotaza, [^125^I]IBETA, [^125^I]IPPI and [^124^I]IPPI binding. Binding in the corpus callosum was subtracted from the anterior cingulate to give a measure of the specific binding of each radiotracer. These specific binding measurements from each subject for the different radiotracers were then related to IHC.

### 2.8. QuPath Image Analysis

Using QuPath, a pixel threshold was created to outline the IHC images. Several annotations were made for Aβ plaques and Tau in the grey matter regions of the IHC brain slices of each subject. Approximately 20–25 annotations were made for Aβ plaques or Tau by means of visual identification of being an Aβ plaque or an NFT. Negative annotations (approx. 15) with no Aβ plaques or Tau were drawn in each subject. The pixel classifier was run (using either random trees or artificial neural networks), and the entire brain slice of each subject was generated by the pixel classifier and saved as a new image. To measure the area of Aβ plaques or Tau for each new pixel-classified brain slice, regions of interest (ROI) for grey matter and white matter were drawn on the new classified image. This ROI was then run through the classifier again and asked to measure all annotations. Area of Aβ plaques or Tau in the ROI’s, total area and percent of Aβ or Tau were obtained for each brain slice. This percent positivity of either Aβ plaques or Tau was used to correlate with autoradiography measures.

## 3. Results

### 3.1. [^124^I]IPPI

Electrophilic radiolabeling with iodine-124 of the tributyltin precursor was efficient. The purification and isolation of [^124^I]IPPI using preparative HPLC provided the product with a purity of >95% in a final 10% radiochemical yield. Ethanolic solution of [^124^I]IPPI was stable and used for in vitro studies. The stability of [^124^I]IPPI was similar to our previous findings of [^125^I]IPPI [9].

**Figure 5:**
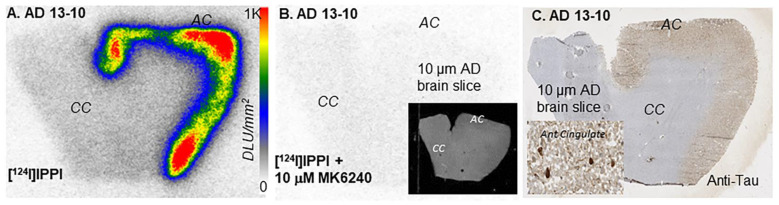
(**A**). Postmortem human brain autoradiography of 10 μm AD brain slice with [^124^I]IPPI showing binding to Tau in anterior cingulate (AC). (**B**). AD brain slice with [^124^I]IPPI + MK-6240 showing blocking effect of 10 μM MK-6240 in AC (inset shows scan of slice). (**C**). Immunostaining of brain slice with anti-Tau for total Tau of entire slice (inset at 20 μm showing Tau).

Our preliminary studies with [^124^I]IPPI using AD anterior cingulate sections show excellent binding in Tau-rich regions with low levels of nonspecific binding in the corpus callosum. (Figure 5A). The ratio of anterior cingulate to corpus callosum binding of [^124^I]IPPI was >10. This high level of binding was greater than that observed for [^125^I]IPPI. Because of the higher energy emission from iodine-124 compared to iodine-125, a shorter exposure time (24hrs) is sufficient to obtain high-quality images, as seen in Figure 5A. It should be noted that alcohol used in the washing procedures enabled the low levels of nonspecific binding seen in the corpus callosum. The binding of [^124^I]IPPI was blocked by the known Tau agent, MK-6240, as seen in Figure 5B and previously shown for [^125^I]IPPI [9]. The complete displacement of [^124^I]IPPI by MK-6240 confirmed the binding of [^124^I]IPPI to the Tau sites found in the AD brain, similar to the PET imaging agent [^18^F]MK-6240 [14]. Adjacent brain sections’ IHC staining confirmed the presence of Tau, and the binding of [^124^I]IPPI correlated well with the IHC-stained anterior cingulate (Figure 5C). The absence of Tau in the corpus callosum was confirmed in the IHC (Figure 5C).

### 3.2. Aβ Plaques Human Anterior Cingulate

**Figure 6:**
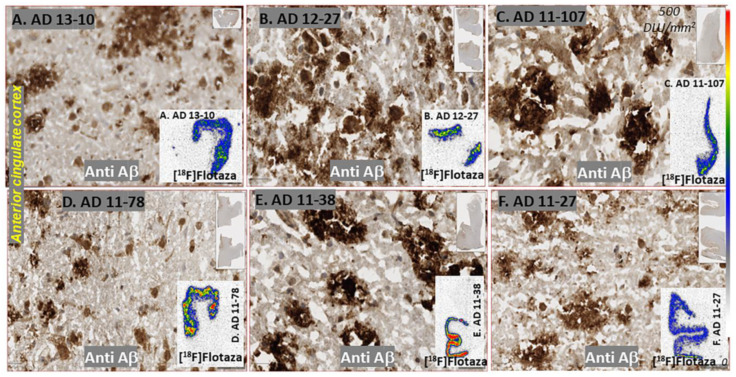
AD subjects Anti Aβ: Anterior cingulate of six AD subjects IHC stained with anti-Aβ at 20 μm resolution. All subjects show extensive neuritic, cored and diffuse plaques. Subjects showed varying amounts of Aβ plaque positivity (4 to 15%). Insets in (**A**–**F**) show extensive binding of [^18^F]flotaza to Aβ plaques in the anterior cingulate (blue–green–red regions show grey matter regions of anterior cingulate with high Aβ plaque content).

**Figure 7:**
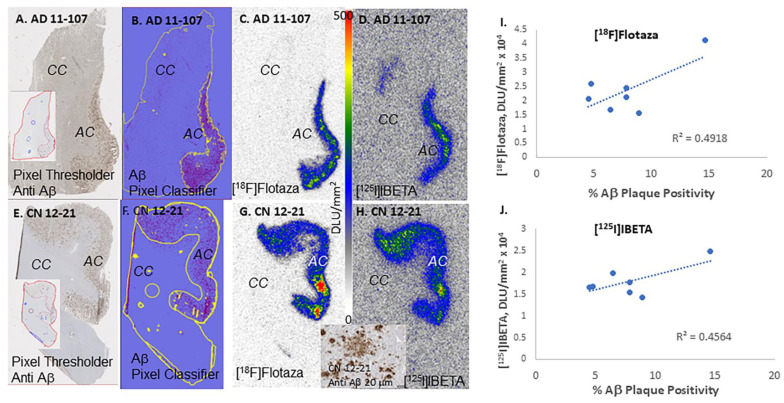
Aβ plaques in AD and control subjects: (**A**)**.** Anti-Aβ IHC (Inset shows pixel threshold of AD 11–107); (**B**). Aβ pixel classifier image of AD 11–107; (**C**). [^18^F]Flotaza binding to anterior cingulate (AC) of AD 11–107 with no binding to corpus callosum (CC); (**D**). [^125^I]IBETA binding to anterior cingulate (AC) of AD 11–107 with no binding to corpus callosum (CC); (**E**). Anti-Aβ IHC (Inset shows pixel threshold of CN 12–21); (**F**). Aβ pixel classifier image of CN 12–21; (**G**). [^18^F]Flotaza binding to anterior cingulate (AC) of CN 12–21 with no binding to corpus callosum (CC); (**H**). [^125^I]IBETA binding to anterior cingulate (AC) of CN 12–21 with no binding to corpus callosum (CC); (Inset shows anti Aβ IHC at 20 μm); (**I**). Correlation plot of % Aβ plaque positivity and [^18^F]flotaza binding in 6 AD and 1 CN subject; (**J**). Correlation plot of %Aβ plaque positivity and [^125I^]IBETA binding in 6 AD and 1 CN subject.

Immunostains of the six AD subjects revealed the presence of extensive amounts of diffuse and neuritic Aβ plaques (Figure 6). The Aβ plaques were uniformly distributed throughout the anterior cingulate regions in all the subjects, while the corpus callosum was devoid of Aβ plaques, as expected. Using QuPath, annotations were made on the Aβ plaques present in the anterior cingulate regions of each subject seen in Figure 6. Negative regions were used in the corpus callosum where no Aβ plaques were present. These annotations were used as the pixel thresholds, and the pixel classifier was used to identify Aβ positivity in the anterior cingulate of each subject, as described in Figure 1. The percentage of Aβ positivity ranged from 4% to 15% across the six AD subjects. This high positivity is evident from the images (20 μm) seen in Figure 6. Among the control subjects (Table 1), only one subject had high levels of Aβ plaques. The levels of Aβ plaques in this control subject were comparable to those found in AD subjects by IHC (Figure 7E). Analysis by QuPath revealed an Aβ positivity of 8% in the anterior cingulate, which falls at the lower end of positivity observed in the AD subjects. It was also noted that the control subject had greater amounts of diffuse plaques with fewer neuritic plaques compared to the AD subjects (inset in Figure 7G).

Binding of [^18^F]flotaza to Aβ plaques was seen in the anterior cingulate regions of the six AD subjects and one CN subject. Figure 6 insets show brain slices of all six subjects with binding of [^18^F]flotaza in the anterior cingulate, with very little white matter binding. The high levels of [^18^F]flotaza binding were consistent with immunostaining in adjacent sections. Because of the differences in resolution between the two modalities (IHC and autoradiography, μm^2^ versus mm^2^), a closer micron-level assessment of [^18^F]flotaza binding may not be possible. However, using QuPath analysis of Aβ positivity of the anterior cingulate region (pixel classifier in Figure 7B) and [^18^F]flotaza binding in the same region (Figure 7C), a correlation plot was examined (Figure 7I). The diffuse plaques in the control subject are shown in Figure 7E, which were used to develop the pixel classifier image in Figure 7F. High levels of [^18^F]flotaza were observed in this control subject, as shown in Figure 7G,H. Since white matter binding was very small across all the AD and CN subjects, ratios between anterior cingulate and corpus callosum were found to be >70 in all the subjects. The correlation plot of percent Aβ positivity and specific binding of [^18^F]flotaza in six AD and one control subject is shown in Figure 7I. Since the AD subjects used in the present study are advanced cases (Table 1) with a high level of Aβ plaques, a plateauing effect of [^18^F]flotaza is seen. The high levels of [^18^F]flotaza binding seen in the control subject suggest that [^18^F]flotaza binds to diffuse plaques equally well. The [^18^F]flotaza ratio of anterior cingulate to corpus callosum in the control subject was >30.

The binding of the radioiodinated Aβ plaque imaging agent, [^125^I]IBETA [11], was evaluated in all the subjects. Our previous studies of displacement of [^125^I]IBETA by flotaza in the human postmortem AD brain [11] and the model studies reported here (Figure 3) confirm that the binding sites of both flotaza and IBETA are similar. The binding of [^125^I]IBETA in all the six AD subjects and one control subject was high in the anterior cingulate (AC/CC = 20), similar to our findings with [^18^F]flotaza. However, the ratio of anterior cingulate to corpus callosum for AD subjects using [^125^I]IBETA was lower (~40) compared to [^18^F]flotaza, as seen in Figure 7. Anti-Aβ immunostaining correlated with [^125^I]IBETA regional binding as shown in Figure 7B,D,F,H. There was a correlation between the binding of [^125^I]IBETA and anti-Aβ in the anterior cingulate. Additionally, the binding of [^18^F]flotaza and [^125^I]IBETA were also correlated, confirming the similarity of binding.

### 3.3. Tau Human Anterior Cingulate

**Figure 8:**
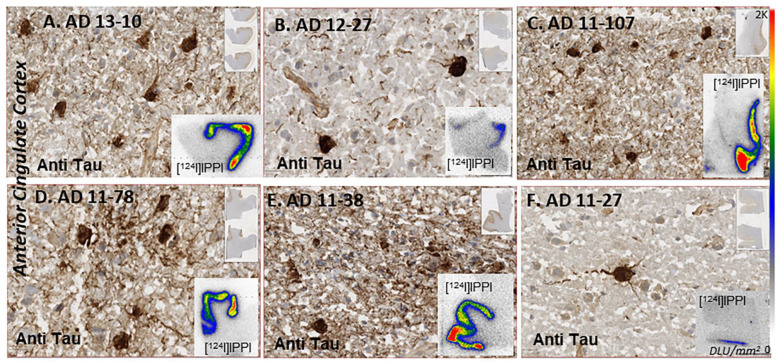
Anti-Tau: Anterior cingulate of six AD subjects IHC stained with anti-Tau at 20 μm resolution. Subjects showed varying amounts of Tau positivity (1.3 to 35%). Insets in (**A**–**F**) show binding of [^124^I]IPPI to Tau in the anterior cingulate.

Tau immunostains of the six AD subjects revealed the presence of varying amounts of tangles, as shown in Figure 8. Tau was distributed throughout the anterior cingulate regions in all the subjects, while the corpus callosum was devoid of any Tau. One of the AD subjects (11–27) had very sparse Tau IHC. None of the control subjects exhibited any presence of Tau. This included the one subject CN 12–21 who had exhibited the presence of extensive amounts of Aβ plaques (Figure 7G,H).

Using QuPath, annotations were made on the Tau present in the anterior cingulate regions of each subject seen in Figure 8. Negative regions were used in the corpus callosum where no Tau was present. These annotations were used as the pixel thresholds, and the pixel classifier was used to identify Tau positivity in the anterior cingulate of each subject, as described in Figure 1. The percentage of Tau positivity ranged from 1.3% to 35% across the six AD subjects. This range of Tau positivity is evident from the images (20 μm) seen in Figure 8.

**Figure 9:**
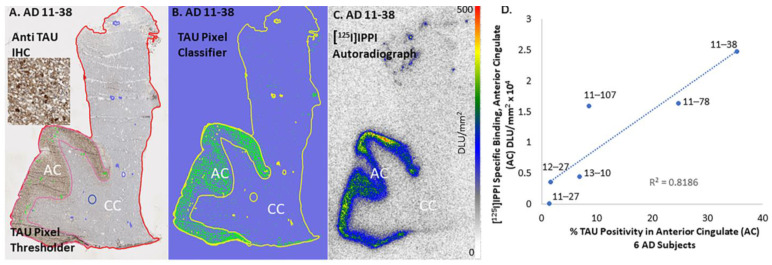
Tau in human anterior cingulate (AC) and corpus callosum (CC): (**A**). Anti-Tau IHC showing NFT in the gray matter (GM) regions with little or no staining in white matter (WM) regions; (**B**). Pixel threshold and pixel classifier identify all Tau positivity in GM and WM regions; (**C**). [^125^I]IPPI Tau, DLU/mm^2^ in GM and WM regions; (**D**). Correlation plot of [^125^I]IPPI per mm^2^ with % Tau positivity.

Binding of [^124^I]IPPI and [^125^I]IPPI to Tau was seen in the anterior cingulate regions of all six AD subjects. Figure 8 insets show brain slices of all six subjects with binding of [^124^I]IPPI in the anterior cingulate, with very little white matter binding. The high levels of [^124^I]IPPI binding were consistent with immunostaining in adjacent sections. Because of the differences in resolution between the two modalities (IHC and autoradiography, μm^2^ versus mm^2^), a closer micron-level assessment of [^124/125^I]IPPI binding may not be possible. However, using QuPath analysis of Tau positivity of the anterior cingulate region (pixel classifier seen in Figure 9A,B) and [^125^I]IPPI binding in the same region (Figure 9C), a correlation plot was examined (Figure 9D). Tau positivity ranged from low (<2%) to over 30%. The correlation plot of percent Tau positivity and specific binding of [^125^I]IPPI in six AD is shown in Figure 9D. Although the AD subjects used in the present study were advanced cases (Table 1), a significant linear positive correlation of [^125^I]IPPI was observed. All control subjects exhibited little binding of [^125^I]IPPI in the anterior cingulate and corpus callosum. Because of the higher photon energy of iodine-124 compared to iodine-125, the image quality of [^124^I]IPPI was superior to [^125^I]IPPI.

## 4. Discussion

Molecular biomarkers for AD are now indispensable for the clinical definition of the process and stage of the disease [28]. Efforts have been underway on the development and use of Aβ plaque and Tau PET imaging agents [29]. Consistent with CSF measures of various forms of Tau, non-invasive imaging can play an essential role in clinical studies for the evaluation of disease progression [3]. Direct measures of brain biomarkers for earlier diagnosis need to be pursued in order to understand the stage of the disease. During the course of development of the radiotracers for the two biomarkers, in vitro autoradiographic evaluation is carried out in postmortem brain tissue, and qualitative confirmation of the binding to Aβ plaques or Tau with IHC is used. Our findings reported here suggest that a more quantitative comparison of autoradiography and IHC provides a confirmation with greater certainty of the variations of radiotracer autoradiography within and across different subjects. Furthermore, this methodology may be useful to establish changes in the biomarkers with disease progression.

Several fluorine-18-labeled PET Aβ-amyloid plaque radiotracers for Alzheimer’s disease (AD) are in clinical use. For PET imaging, we have recently developed [^18^F]flotaza [10] and radioiodinated [^124/125^I]IBETA [11]. Molecular docking evaluation of flotaza and IBETA to the amyloid fibril revealed a preference for a similar binding site (Figure 3). The possibility of small molecule binding to several different sites in the amyloid fibril has been reported [30]. In the case of flotaza and IBETA, the similarity of the binding site has been further confirmed by in vitro competition experiments in postmortem human brains [11]. Since Aβ plaques may be seen in a diffuse, opaque or neuritic forms in the AD brain [31], preservation and accessibility of the amyloid fibril structure are necessary for the radiotracers to bind in order to enable diagnosis. The six AD subjects reported here had an abundance of opaque and neuritic plaques, and all of them exhibited high binding of [^18^F]flotaza and [^125^I]IBETA. The control subject with high levels of [^18^F]flotaza and [^125^I]IBETA binding appeared to have greater levels of diffuse plaques (Figure 7). It may thus be surmised that the amyloid fibril structure is preserved in the diffuse, opaque and neuritic plaques and that the radiotracers [^18^F]flotaza and [^125^I]IBETA are able to bind to them. Although it would be useful to identify this binding at the higher 10–20 μm resolution in the IHC images (Figure 6), the autoradiography image resolution is 10^2^–10^3^ times lower.

Using QuPath analysis on all the anti Aβ positive brain sections (6 AD subjects and 1 CN subject), the percent Aβ plaque positivity in the anterior cingulate ranged from approximately 4% to 15%. Within each subject's brain sections, there was a good correlation between the binding of [^18^F]flotaza and anti-Aβ IHC. Due to the very low nonspecific binding of [^18^F]flotaza in the corpus callosum, the sensitivity of [^18^F]flotaza to measure the presence of Aβ plaques was very high, as seen in Figure 6 and Figure 7. A correlation plot between [^18^F]flotaza binding and percent Aβ positivity for the seven subjects are shown in Figure 7I. A moderate positive correlation was observed. This may be due to the limited number of subjects with advanced AD. Aside from one high binder, the other subjects appeared to reach a threshold of [^18^F]flotaza binding in the four to nine percent Aβ positivity. The same subjects were evaluated using [^125^I]IBETA, and the corresponding images are shown in Figure 7D,H. Because of the iodine-125 radioisotope and the physical properties of [^125^I]IBETA, the background activity was higher. A correlation plot between [^125^I]IBETA binding and percent Aβ positivity for the seven subjects in Figure 7J was similar to that observed with [^18^F]flotaza. Efforts are currently underway on differentiating diffuse, opaque and neuritic plaques using QuPath. This will help understand the binding properties of both [^18^F]flotaza and [^125^I]IBETA to the different forms of Aβ plaque forms. It is expected that the fibril binding sites shown in Figure 3 may be conserved in the different plaque forms. However, this needs to be ascertained.

The ability to quantitatively assess the percent of Aβ plaque positivity in the mouse model will be useful in imaging studies of other neurotransmitter–receptor systems in the AD mouse models [32]. These approaches may also be applied to α-synucleinopathies [33,34,35]. Our [^124^I]IBETA PET/CT studies in the 5×FAD mouse model showed greater localization of [^124^I]IBETA in the brain regions with a high concentration of Aβ plaques [11]. Therefore, [^124^I]IBETA may potentially be a useful PET radioligand for Aβ plaques in brain studies.

There is now increased focus on Tau as a more accurate, early predictive marker for AD diagnosis. PET imaging studies with [^18^F]MK-6240 have been underway in AD. Development of Tau imaging agents continues because of issues of off-target binding [36], as well as a need for more selective Tau imaging agents. We developed [^125^I]IPPI for Tau imaging of human postmortem AD [9]. The binding of IPPI on the Tau model revealed four binding sites similar to MK-6240 [9]. The binding energies of IPPI for the four sites were approximately similar. The availability of iodine-124 labeled PET radiopharmaceuticals will allow flexibility of transportation to remote PET sites. Because of its longer half-life, iodine-124 will allow for extended imaging times, enabling greater clearance of nonspecific binding and thus providing potentially better image contrast which may be useful when small levels of Tau are present. Our results show that [^124^I]IPPI can be efficiently prepared and is stable for in vitro studies. Binding to Tau in the AD brains gave excellent contrast between anterior cingulate and corpus callosum (Figure 8, insets) and was consistent with the presence of Tau confirmed by anti-Tau IHC. The higher energy iodine-124 photon may also be useful in detecting lower levels of Tau in patients at lower Braak stages. The other iodine radioisotope of interest for single photon emission computed tomography (SPECT) imaging is iodine-123. No iodine-123 radiolabeling and in vivo imaging evaluation have been reported for Tau. The availability of SPECT imaging agents for Tau will allow nuclear medicine clinics, which may not have PET capability, to purchase the iodine-123 labeled radiopharmaceutical for SPECT imaging studies. Thus future studies will include the preparation and evaluation of [^123^I]IPPI as a potential candidate for SPECT imaging.

Of the 12 subjects (6 AD and 6 CN), the AD subjects were positively IHC stained with anti-Tau (Figure 8). None of the control subjects had any Tau, including CN 12–21, who had significant levels of Aβ plaques. Subject AD 11–27 had the lowest amount of IHC-stained Tau (Figure 8F). Analysis of Tau by QuPath classifier confirmed the low levels (1.3%) of Tau positivity in AD 11–27, whereas AD 11–38 had the highest Tau positivity (35%). The high Tau positivity in AD 11–38 (Figure 9B) also resulted in a high degree of [^125^I]IPPI binding (Figure 9C) and [^124^I]IPPI (Figure 8E). A good positive correlation between percent Tau positivity and [^125^I]IPPI was observed (Figure 9D). The detection of Tau in lower Braak stages is more challenging. It may be assumed that all four binding sites for IPPI binding (Figure 4) are present in the lower Braak stages (< IV). It should be noted that anti-Tau measured total Tau. Although both total Tau and phosphorylated Tau (pTau) levels in AD are elevated [18], a correlation of [^125^I]IPPI with pTau positivity may yield stronger. Studies suggest that pTau can quantify longitudinal changes in Tau pathology, identify neurodegeneration and predict AD progression [37]. Higher correlations between p-tau217 and [^18^F]flortaucipir were corroborated in an independent trial cohort [38].

The small number of subjects in advanced stages of AD is a limitation of this study. Comparison of Aβ plaques and Tau in this limited cohort indicates that all AD subjects (including one control) exhibited Aβ plaque positivity (both using IHC and autoradiography) and Tau positivity correlated with autoradiography. Future studies will include more subjects in earlier stages of AD and mild cognitive impairment (MCI). This will allow a better correlation of Aβ plaque and Tau positivity with the corresponding radiotracers, [^18^F]flotaza, [^125^I]IBETA and [^125^I]IPPI.

## 5. Conclusions

Autoradiography binding profile of [^18^F]flotaza, [^125^I]IBETA and [^125^I]IPPI to Aβ plaques and Tau and the correlation with IHC revealed the quantitative relationship between the two modalities. This method allows a more comprehensive analysis of Aβ plaques and Tau in a given brain slice and across subjects. Compared to semi-quantitative histopathological scoring, our methods of analyzing IHC with radiotracer autoradiography binding provides a quantitative method of image analysis.

## Figures and Tables

**Table 1:** Patient samples and data ^1^.

ID	Pathology	Gender	Age Expired	Braak Score	Plaque Total ^2^	Tangle Total ^2^	[^18^F]Flotaza Aβ ^3^	[^125^I]IBETA Aβ ^4^	[^125^I]IPPI Tau ^5^
10–39	CN	Male	93	I	0	1	0	0	0
10–63	CN	Male	79	II	0	2.5	0	0	0
10–70	CN	Male	74	I	0	2	0	0	0
13–40	CN	Male	73	II	0	2.25	0	0	0
13–49	CN	Female	75	II	0	2.5	0	0	0
12–21	CN ^6^	Female	88	II	14	3.5	++	++	0
11–107	AD	Male	75	VI	14	15	+++	++	+++
11–27	AD	Male	78	V	12.5	10	++	++	+
11–38	AD	Male	76	VI	14.5	15	++++	+++	+++
11–78	AD	Male	89	V	14.5	15	+++	++	+++
13–10	AD	Male	78	VI	14	14	+++	++	++
12–27	AD	Female	86	VI	15	15	+++	++	++

^1^ Frozen brain samples of anterior cingulate cortex and corpus callosum were obtained from Banner Sun Health Research Institute (BHRI), Sun City, Arizona [2]; CN = cognitively normal and may include mild cognitive impairment (MCI) subjects; AD = Alzheimer’s disease; ^2^ Plaque total: Includes neuritic, cored and diffuse, in frontal, temporal, parietal, hippocampal and entorhinal cortex. Semi-quantitative scores of none, sparse, moderate and frequent were converted to numerical values 0–3 for each region and summed to provide Plaque total; Tangle total: neurofibrillary tangle density in frontal, temporal and parietal lobes, hippocampal CA1 region and entorhinal cortical regions. Numerical values 0–3 for each region were summed to provide Tangle total from different brain regions of the subjects obtained from BHRI. ^3^ [^18^F]flotaza, measured as Digital light units (DLU)/mm^2^ ++ (<20 × 10^3^ DLU/mm^2^), +++ >20 × 10^3^ DLU/mm^2^), ++++ >40 × 10^3^ DLU/mm^2^); ^4^ IBETA ++ (<20 × 10^3^ DLU/mm^2^), +++ >20 × 10^3^ DLU/mm^2^) ^5^ Tau + (<2 × 10^3^ DLU/mm^2^), ++ (<10 × 10^3^ DLU/mm^2^), +++ (>20 × 10^3^ DLU/mm^2^) ^6^ Microscopic changes of AD, insufficient for AD diagnosis.

## Data Availability

The data that support the findings of this study are available from the corresponding author upon reasonable request.
